# Barriers in Implementing E-Learning in Hormozgan University of Medical Sciences

**DOI:** 10.5539/gjhs.v8n7p83

**Published:** 2015-11-03

**Authors:** Parvin Lakbala

**Affiliations:** 1 Health Information Management Research Center, Hormozgan University of Medical Sciences, Bandar Abbas, Iran; 2 Department of Health Information Technology, Faculty of Para-Medicine, Hormozgan University of Medical Sciences, Bandar Abbas, Iran

**Keywords:** E-learning, technology, learning, distance

## Abstract

**Background:**

E-learning provides an alternative way for higher educational institutes to deliver knowledge to learners at a distance, rather than the traditional way. The aim of this study is to identify the barrier factors of e-learning programs in Hormozgan University of Medical Sciences (HUMS) in respect of the students and lecturers’ point of view.

**Methods:**

A cross-sectional study based on a questionnaire was conducted among 286 of students and lecturers in the nursing, midwifery and paramedic schools of HUMS. Two hundred and eighty-six participants filled in the questionnaire: 256 students, and 30 lecturers.

**Results:**

Results of the study showed a lack of proper training in e-learning courses of the university 182 (69.1%), limited communication with the instructor 174 (68%) and the learners dominance of English language 174 (68%) showed the greatest importance for the students. The awareness about e-learning program was 80% and 43% among lecturers and students respectively.

The dominance of English language 26 (86.7%) and lack of research grants for e-learning 23 (76.6%) and lack of proper training on e-learning courses from the university 20 (66.7 %) were the most important barrier factors of implementing e-learning for lecturers. E-learning courses to supplement classroom teaching was a solution that mentioned by the majority of students 240 (93.8%) and lecturers 29 (96.7%) in this study.

**Conclusions:**

The positive perception of e-learning is an important consequence effect in the future, educational development of nursing, midwifery and paramedic schools.

## 1. Background

The Internet is now widely used as a communication medium for personal, commercial and educational purposes. E-learning is rapidly becoming a key element of institutional teaching and learning strategies with many academic departments seizing the opportunity to use new technologies to enhance their educational provision. Commonly, universities support it through the provision of virtual learning environments which provide students with access to single and multi-media course materials, online collaboration and computer aided assessment.

Information and Communication Technology (ICT) has become a major focus of interest in the educational field. There are many benefits which speak for the integration of ICT in education, such as increasing the quality of learning (Chang, 2006), providing learners with technological skills and encouraging learners to be more interactive (Van-Braak, 2001), promoting teachers and students’ performance and motivation, and removing the limitations of time and space in instructional processes (Al-zaidiyeen et al., 2008).

Functionally e-learning includes a wide variety of learning strategies and ICT applications for exchanging information and gaining knowledge. Such ICT applications include television and radio; Compact Discs (CDs) and Digital Versatile Discs (DVDs); video conferencing; mobile technologies; web-based technologies; and electronic learning platforms. This section discusses what these ICTs entail as well as their pedagogical, technical and cost implications (Sife, 2007).

The web conferencing technologies integrate presentation, collaboration and communication on one platform. Providing synchronous online learning space they offer possibility of emulation of traditional classrooms (Morningstar & Primlani, 2006). The role of modern teacher has changed. He is now not only the source of knowledge, but also the activator of discussion and a stimulator of creativity and critical thinking (Hernandez et al., 2007).

While the value of e-learning lies in its ability to train anyone, anytime, anywhere, implementing and sustaining e-learning programs require more than merely moving education and learning online (Harris, 2002). Secondly, if we are to develop, deliver and administer e-learning programs, and train educators to become competent e-learning facilitators, a high level of investment in ICT infrastructure is required. Successful e-learning implementation therefore depends on building a strategy that meets the needs of the learners and the business goals of the institution. In Iran among 44 universities of medical sciences only 4 universities have managed to implement the e-learning platform (Mehralborz University, 2015). The aim of this study is to identify lecturers’ and students’ perceptions toward the importance of barriers factors and their attitudes toward various methods for implementing e-learning program in the Hormozgan University of Medical Sciences (HUMS).

## 2. Methods

A cross-sectional study based on a questionnaire was carried out with two different groups of subjects: students academic group (Nursing and Midwifery, Laboratory, Health information technology, Radiology, Anesthesia and Operation room technology) and lecturers of the paramedic, nursing and midwifery schools of HUMS, Bandar Abbas, Iran. For the academic year 2013–2014, the paramedic, nursing and midwifery schools of HUMS was constituted by approximately 1300 students and 46 lecturers. Stratified sampling method was used which included 346 respondents representing a variety of two different groups (lecturers and students).

Data were collected through two anonymous questionnaire instrument that distributed to 300 students of the paramedic, nursing and midwifery schools of HUMS students and 46 lectures during the fall semester of 2014 where 286 (83%) were returned. Respondents for this study consisted of 256 students (166 females and 90 males) and 30 lectures (15 females and 15 males).

The questionnaire was designed especially for the purpose of this study. It was based on a literature review of previous studies regarding participants’ perceptions toward the Importance of barriers factors and participants’ attitudes toward various methods for implementing e-learning program.

We tested the questionnaire in a pilot study with a sample of 10 students and 2 lecturers who were not a part of the study population (Heath school students and lecturers at HUMS). A few changes in the wording of questions were made as a result of the pilot study.

The questionnaire was designed with 53 questions for lecturers and 43 questions for students. Questions were divided into several sections: Demographic questions, access to computers and the Internet; knowledge and perception of e-learning; participants’ perceptions regarding the Importance of barriers factors and participants’ attitudes toward various methods for implementing e-learning program. Various methods of questions were proposed: multiple choices and Likert scale type questions from ‘very much’ to ‘very little’ was used to determine participants’ perceptions regarding the Importance of barrier factors and ‘strongly agree’ to ‘strongly disagree’ to determine participants’ attitudes toward various methods for implementing e-learning program. Students’ perceptions were evaluated based on a scale ranging from “very much” to “very little” in the Six categories of barrier factors (access-skill barriers, attitudinal barriers, cultural barriers, infrastructural barriers, barriers associated with integrating e-learning with traditional teaching and Disproportion between method and content) with 20 sub- categories. Lecturers perceptions were evaluated based on a scale ranging from “very much” to “very little” in Seven categories of barrier factors (access-skill barriers, attitudinal barriers, cultural barriers, infrastructural barriers, barriers associated with integrating e-learning and traditional teaching, disproportion between method and content credit &encouraging barriers) and 24 sub- categories.

### 2.1 Ethics

This study was funded by the Deputy of Research and Technology of Hormozgan University of Medical Sciences, Iran (Grant No. 9381). The ethical review committee of Hormozgan University of Medical Sciences did not consider this study to require approval. Informed consent was obtained from participants.

### 2.2 Data Analysis

The collected data were analyzed using SPSS v. 12 (SPSS, Chicago, IL, USA) and χ^2^ test, with P < 0.05 considered statistically significant. The percentages and their 95% confidence intervals are presented.

## 3. Results

### 3.1 Response rates

Out of 346 questionnaires which distributed (300 for students and 46 for lecturers), 286 (83%) questionnaires were completed and analyzed (256 from the students and 30 from the lecturers).

### 3.2 Participant Profile

All students were at the bachelor level. The group of lecturers came from 7 academic departments out of whom 56.9 had a long teaching experience (10 to over 20 years) (Table 1).

**Table 1:** Participant profile

Participant profile	No. (%)
**Gender**	
Lecturers:	
Male	50
Female	50
**Students**	
Male	37
Female	63
**Age group**	
Lecturers:	
<30	20
30-39	36.7
40-49	33.3
>50	10
Students:	
19-24 years	82
25-29 years	9
>30	9
**Lecturers educational level**	
Master degree	83.3
Ph.D.	16.7
**Lecturers years work**	
<10	43.3
10-20	23.3
>20	33.4
**Academic department (students):**	
Laboratory	18.8
Health Information Technology	11.9
Radiology	9.8
Anesthesia	14.1
Operating room	16
Nursing	13.7
Midwifery	16

### 3.3 Knowledge About E-Learning and Computer

Results showed that the use of computers and Internet among students and lecturers were between 1 to 8 hours per day. The awareness about e-learning program was 80% among lecturers, but 66.7% of them announced that they had not received an e-learning program in the past. Among lecturers, 40% of them were aware of the barrier factors for the implementation of distance education programs at the university. Among student group 21.5% of them had a private web and 26.6% had the experience in writing web. Awareness about e-learning program between student groups was 43% between student groups. Among students, 32.8% of them had received e-learning program in the past.

### 3.4 E-Learning Barrier Factors

Table 2 summarizes the students’ perceptions regarding the importance of barrier factors to the implementation of e-learning in university. Pedagogical, technical and cost issues should be taken into account each specific technology while integrating ICTs in teaching and learning practices.

**Table 2:** Students’ perceptions associated with the importance of barrier factors for the implementation of e-learning

Students’ Barrier factors	Very much (%)	Much (%)	Moderate (%)	Little (%)	Very little (%)
**Access-Skill Barriers**					
Low access to computer	94(36.7)	68(26.6)	58(22.7)	28(10.9)	8(3.1)
Inadequate computer	54(21.1)	99(38.7)	74(28.9)	18(7.0)	11(4.3)
Little computer knowledge of students	62(24.2)	89(34.8)	75(29.3)	27(10.5)	3(1.2)
Limited communication with the instructor	66(25.8)	108(42.2)	63(24.6)	14(5.4)	5(2.0)
Poor communication	45(17.6)	97(37.9)	86(33.6)	20(7.8)	8(3.1)
**Attitudinal barriers**					
Lack of interest in e-learning	40(15.6)	77(30.1)	85(33.2)	37(14.5)	17(6.6)
No need for E-Learning	39(15.2)	77(30.1)	76(29.7)	43(16.6)	21(8.2)
Negative attitudes towards new technologies	38(14.8)	54(21.1)	74(28.9)	57(22.3)	33(12.9)
**Cultural Barriers**					
Lack of support	54(21.1)	92(35.9)	67(26.2)	25(9.8)	18(7.0)
Computer knowledge not require	42(16.4)	86(33.6)	68(26.6)	42(16.4)	18(7.0)
Lack of comfort with technology Concerns about the ethical issues in the use of Internet	36(14.1)	76(29.7)	67(26.2)	52(20.3)	25(9.8)
	43(16.8)	69(27.0)	73(28.5)	51(19.9)	20(7.8)
**Infrastructure barriers**					
Limited infrastructure to support	61(23.8)	97(37.9)	67(26.2)	23(9.0)	8(3.1)
Lack of proper training in e-learning courses	90(32.2)	92(35.9)	49(19.1)	19(7.4)	6(2.3)
High cost of establishing	57(22.3)	81(31.6)	85(33.2)	25(9.8)	8(3.1)
**Barriers associated with integrating e-learning and traditional teaching**					
Complexity of integrating e-learning with classroom instruction					
Learners mastery to English language	43(16.8)	94(36.7)	82(32.0)	28(10.9)	9(3.5)
					
	74(28.9)	100(39.1)	59(23.0)	19(7.4)	4(1.6)
**Disproportion between method and content**					
Disproportion of e-learning with curriculum content	45(17.6)	105(41.0)	74(28.9)	24(9.4)	8(3.1)
Disproportion of e-learning courses for the academic mission					
Concerns about the practical nature of some courses are not offered electronically	35(13.7)	99(38.7)	80(31.2)	33(12.9)	9(3.5)
					
	40(15.6)	90(35.2)	86(33.6)	31(12.1)	9(3.5)

Lack of proper training in e-learning courses in the university 182 (69.1%), limited communication with the instructor 174 (68%) and the learners dominance of English language 174 (68%) showed the greatest importance for the students (Table 2). Result showed negative attitudes towards new technologies 92 (35.9%). Lack of comfort with technology, ranked the least important among students as the barrier factor for the implementation of e-learning programs. There are, indeed, significant associations between the field of study and lack of interest in e-learning program (χ^2^ = 53.505, df =28, P < 0.05) and not requiring knowledge of computer (χ2 = 52.854, df = 28, *P* <0.05) with students’ perceptions (table 2). Lack of guidance and information prior to enrollment in e-learning, perceived lack of support from faculty, and difficulties in contacting them mentioned in prior studies (Brown, 1996; Pierrakeas et al., 2004; Tresman, 2002). Other researchers have found that student characteristics such as computer literacy and confidence, reading ability, and time management skills played a role in successful course completion (Miller et al., 2003, Osbon, 2001; Rovai, 2003). Student skills in their comprehension of the English language and lack of knowledge of the software are important factors in successful implementation of e-learning course.

Table 3 summarizes the lecturers’ perceptions regarding the importance of barrier factors to the implementation of e-learning in university. Regarding the lecturers’ perceptions in this survey, the dominance of English language 26 (86.7%), lack of research grants for e-learning 23 (76.6%) and lack of proper training on e-learning courses from the university 20 (66.7 %) were the most important barrier factors for the implementation of e-learning.

**Table 3:** Lecturers’ perceptions associated with the importance of barrier factors for the implementation of e-learning

Lecturers’ Barrier factors	Very much (%)	Much (%)	Moderate (%)	Little (%)	Very little (%)
**Access-Skill Barriers**					
Low access to computer	10(33.3)	11(36.7)	6(20.0)	2(6.7)	1(3.3)
Inadequate computer	5(16.7)	12(40.0)	6(20.0)	6(20.0)	1(3.3)
Little computer knowledge of students	5(16.7)	9(30.3)	11(36.7)	2(6.7)	3(10.0)
Limited communication with the instructor	5(16.7)	11(36.7)	13(43.3)	1(3.3)	-
Poor communication	6(20.0)	11(36.7)	4(13.3)	7(23.3)	2(6.7)
**Attitudinal barriers**					
Lack of interest in e-learning	3(10.0)	11(36.7)	8(26.7)	7(23.3)	1(3.3)
No need for E-Learning	6(20.0)	10(33.3)	7(23.3)	5(16.7)	2(6.7)
Negative attitudes towards new technologies	3(10.0)	4(13.3)	5(16.7)	13(43.3)	5(16.7)
**Cultural Barriers**					
Lack of support	4(13.3)	12(40.0)	11(36.7)	2(6.7)	1(3.3)
Computer knowledge not require	4(13.3)	11(36.7)	9(30.0)	5(16.7)	1(3.3)
Lack of comfort with technology Concerns about the ethical issues in the use of Internet	3(10.0)	8(26.7)	9(30.0)	9(30.0)	1(3.3)
	4(13.3)	9(30.3)	8(26.7)	7(23.3)	2(6.7)
**Infrastructure barriers**					
Limited infrastructure to support	8(26.7)	14(46.7)	8(26.7)	-	-
Lack of proper training in e-learning courses	6(20.0)	14(46.7)	8(26.7)	2(6.7)	-
High cost of establishing	8(26.7)	7(23.3)	14(46.7)	1(3.3)	-
**Barriers associated with integrating e-learning and traditional teaching**					
Complexity of integrating e-learning with classroom instruction					
Learners mastery to English language	2(6.7)	11(36.7)	15(50.0)	1(3.3)	1(3.3)
					
	20(66.7)	6(20.0)	3(10.0)	-	1(3.3)
**Disproportion between method and content**					
Disproportion of e-learning with curriculum content	5(16.7)	13(43.3)	7(23.3)	5(16.7)	-
Disproportion of e-learning courses for the academic mission					
Concerns about the practical nature of some courses are not offered electronically	4(13.3)	8(26.7)	12(40.0)	51(16.7)	1(3.3)
					
	6(20.0)	7(23.3)	15(50.0)	11(3.3)	1(3.3)
**A credit& encouraging Barriers**					
Lake of research grants for e-learning	10(33.3)	13(43.3)	4(13.3)	31(10.0)	-
Lake of Support from university	11(36.7)	12(40.0)	7(23.3)	-	-
Lake of support from colleagues Lack of professional credentials to teach in e-learning	4(13.3)	12(40.0)	9(30.0)	4(13.3)	1(3.3)
	2(6.7)	11(36.7)	8(26.7)	7(23.2)	2(6.7)

Lecturers noted that negative attitudes towards new technologies 7 (23.3%) and lack of comfort with technology 11 (36.7%) had the lowest degree of importance for the implementation of e-learning program. There are, indeed, significant associations between the lecturers, education level and complexity of integration of e-learning with classroom instruction (χ^2^ = 10.975, df =4, *P* < 0.05) and disproportion of e-learning with curriculum content knowledge of (χ^2^ = 10.937, df = 4, *P* <0.05) and lectures perceptions (Table 3).

Table 4 summarizes the students’ attitude towards solutions for the implementation of e-learning. E-learning courses to supplement classroom teaching was a solution that mentioned by the majority of respondents 240 (93.8%) in this study (Table 4).

**Table 4:** Students’ attitude towards solutions for the implementation of e-learning

Variables	Strongly Agree (%)	Agree (%)	No comment (%)	Disagree (%)	Strongly Disagree (%)
E-learning courses to supplement classroom teaching	142(55.5)	98(38.3)	14(5.5)	-	2(0.8)
E-learning courses jointly with local universities	116(45.3)	114(44.5)	23(9.0)	1(0.4)	2(0.8)
E-learning courses jointly with foreign universities	112(43.9)	95(37.1)	45(17.6)	4(0.6)	-
Centralized e-learning courses in the centers and units	84(32.8)	120(46.9)	4(16.0)	6(2.3)	5(2.0)
Two-way pattern (e-learning and traditional courses)	66(25.8)	95(37.1)	79(30.9)	10(3.9)	6(2.3)
Collaboration with companies in the field of e- learning	87(34.0)	103(40.2)	50(19.5)	14(5.5)	2(0.8)
A combination pattern (use some of lessons e-learning)	72(28.1)	95(37.1)	59(23.0)	26(10.2)	4(1.6)
Optional combination pattern (to choose e-learning or lesson traditional by students)	109(42.6)	85(33.2)	46(18.0)	13(5.1)	3(1.2)
E-learning courses as an independent unit and Schools	93(36.3)	98(38.3)	51(19.9)	11(4.3)	3(1.2)
E-learning courses for died serving courses	61(23.8)	69(27.0)	88(34.4)	32(12.5)	6(2.3)
E-learning courses for additional training	45(17.6)	81(31.6)	81(31.6)	37(14.5)	12(4.7)
One-way pattern (courses only in electronic form)	47(18.4)	68(26.6)	77(30.1)	45(17.6)	19(7.4)

Table 5 summarizes Lecturers’ attitude towards solutions for the implementation of e-learning. E-learning courses to supplement classroom teaching was a solution that mentioned by the majority of lecturers 29 (96.7%) in this study (Table 5).

**Table 5:** Lecturers’ attitude towards solutions for the implementation of e-learning

Variables	Strongly Agree (%)	Agree (%)	No comment (%)	Disagree (%)	Strongly Disagree (%)
E-learning courses to supplement classroom teaching	18(60.0)	11(36.7)	1(3.3)	-	-
E-learning courses jointly with local universities	17(56.7)	11(36.7)	2(6.7)	-	-
E-learning courses jointly with foreign universities	15(50.0)	13(43.3)	1(3.3)	1(3.3)	-
Centralized e-learning courses in the centers and units	11(36.7)	10(33.3)	8(26.7)	1(3.3) 3(10.0)	-
Two-way pattern (e-learning and traditional courses)	10(33.3)	11(36.7)	6(20.0)	1(3.3)	-
Collaboration with companies in the field of e- learning	10(33.3)	15(50.0)	4(13.3)	1(3.3)	-
A combination pattern (use some of lessons e-learning)	13(43.3)	10(33.3)	5(16.7)	7(23.3)	1(3.3) 2(6.7)
Optional combination pattern (to choose e-learning or lesson traditional by students)	9(30.0)	7(23.3)	5(16.7)		
E-learning courses as an independent unit and Schools	12(40.0)	10(33.3)	7(33.3)	1(3.3)	-
E-learning courses for died serving coursess	8(26.7)	6(20.0)	7(23.3)	9(30.0)	-
E-learning courses for additional training	5(16.7)	4(13.3)	9(30.0)	12(40.0)	-
One-way pattern (courses only in electronic form)	5(16.7)	3(10.0)	5(16.7)	10(33.3)	7(23.3)

## 4. Discussion

Hormozgan province is located in south of Iran with humid and hot weather. Like other universities in developing countries Hormozgan University of Medical Sciences (HUMS) suffer from an insufficient number of members. One of the greatest benefits of e-learning is that it helps Hormozgan province to reduce the dependency on local teaching staff. Thus, through the use of e-learning the problem of staff, insufficient numbers of members can be minimized because the internet allows the design of interactive course-material which is then delivered over the network to the attending students (Clark & Mayer, 2008).

The present study describes student dominance on English language, limited communication with the instructor and lack of proper training in e-learning courses in the university were important barrier factors to the implementation of e-learning in the HUMS according Students’ perceptions. Lack of guidance and information prior to enrollment in e-learning, perceived lack of support from faculty, and difficulties in contacting them mentioned in prior studies (Brown, 1996; Pierrakeas et al., 2004; Tresman, 2002). Other researchers have found that student characteristics such as computer literacy and confidence, reading ability, and time management skills played a role in successful course completion (Miller et al., 2003; Osbon, 2001; Rovai, 2003). Student skills in their comprehension of the English language and lack of knowledge of the software are important factors in successful implementation of e-learning course.

Regarding the lecturers’ perceptions in this study, the dominance of English language, lack of research grants for e-learning and lack of proper training on e-learning courses from the university were the most important barrier factors for the implementation of e-learning. These barriers mentioned in some studies. Cultural challenge in e-world learning is the issue of language, since the majority of internet content is in English language (Barron, 2000; Van Dam & Rogers, 2002; Wilborn, 1999), Non-English speaking individuals may feel that technology has nothing to offer them since they cannot understand the content.

A study conducted by Trentin showed that teachers’ awareness may be developed towards adopting a wide range of Technical Enhanced Learning (TEL) approaches (Trentin, 2006). This process enhanced the online learning environment as teachers became more knowledgeable with technology and its effects on learning outcomes. Training is an incentive for the faculty members use new technology. Faculty members who attended in-service training and workshops are more likely to use and apply new technology (Sife, 2007; Al-Alwani, 2005; Hermans et al., 2008). Lack of training which is related to using e-learning technology can become a major barrier factor in its implementation.

E-learning courses to supplement classroom teaching was a solution that mentioned by the majority of respondents in this study. In the prior studies some researchers had recommended pre-course orientations to help manage students’ expectations and generally prepared them for distance learning (Tresman, 2002; Rovai, 2003; Ludwig & Dunlap, 2003; Rayan, 2001; Scales, 2001; Wojciechowski, 2005). The success of any initiative with the purpose of implementing technology in an educational program depends strongly upon the attitudes of the faculty members involved (Albirini, 2004; Hamidi, 2002; Zhao et al., 2002). The overall attitude of the faculty members reflected toward computer technology directly influenced the extent of computer utilization. In order to achieve a change in teaching practices which results in a more integrated use of technology, there was a need to examine the opinions of the instructors themselves and their beliefs about innovative teaching approaches such as e-learning (Ertmer, 2005). The use of technology in learning environment more likely increases when the instructors’ pedagogical approach to teaching is consistent with selected technology (Zhao et al., 2002). Positive attitude toward ICTs is widely recognized as a necessary condition for their effective implementation. Pedagogical, technical and cost issues should be taken into account for each specific technology while integrating ICTs in teaching and learning practices.

## 5. Conclusion

E-learning is evolving, whether is intended to supplement traditional face-to-face learning or be used to replace traditional learning altogether within organizations Regardless of the purpose, it is paramount that providers’ designer e-learning uses to the client, and pay attention to differences in cultural learning styles and the preferences of its end users. It is important for all stakeholders of the University of Medical Sciences to know the existing ICT facilities and services and their importance in relation to their specific tasks. The positive perception of e-learning is an important result in the future education, development of the faculty of nursing, midwifery and Paramedic School. Furthermore, the development of participant’s computer skills is a key issue. Research showed that human factors such as lack of computer skills, anxiety towards using computer and personal discipline are critical to the success of e-learning. Students and lecturers skills in English language and their knowledge of the software are important for the implementation of e-learning.
